# The Future of Immunotherapy: A 20-Year Perspective

**DOI:** 10.3389/fimmu.2017.01668

**Published:** 2017-11-28

**Authors:** David C. Wraith

**Affiliations:** ^1^Institute of Immunology and Immunotherapy, College of Medical and Dental Sciences, University of Birmingham, Birmingham, United Kingdom

**Keywords:** cancer, autoimmune disease, immunotherapy, cancer vaccines, multiple sclerosis

## Abstract

Immunotherapy is the field of immunology that aims to identify treatments for diseases through induction, enhancement or suppression of an immune response. Immunotherapies designed to instigate or enhance an immune response are considered “activating immunotherapies” while those designed to repress an immune response are “suppressive immunotherapies.” This perspective will focus on two areas of immunotherapy, activating immunotherapies for cancer and suppressive immunotherapies for autoimmunity both of which have seen a resurgence in interest in recent years and are likely to transform the treatment of many human diseases in the next 20 years. Effective immunotherapies for cancer, where the aim is to activate tumor-specific immune responses, will be totally different from those designed to suppress the immune response to self-antigens in autoimmune disease. Furthermore, the reader will appreciate that the degree to which side effects of immunotherapies are acceptable will differ drastically between life-threatening cancers and chronic, debilitating but not necessarily life-threatening autoimmune conditions.

## The Global Health Burden

Improvements in sanitation and effective vaccination are gradually reducing the impact of infectious diseases across the world. The World Health Organisation (WHO) predicts a continuing decline in global mortality resulting from respiratory, perinatal, and other infections, excluding HIV/AIDS. While global mortality due to AIDS has declined rapidly, this rate is not predicted to change dramatically over the coming 20 years (1). By contrast, global mortality due to cardiovascular disease, cancer, non-infectious respiratory disease, and other inflammatory diseases will increase with cardiovascular disease predicted to be the greatest killer followed by malignant neoplasms and chronic obstructive pulmonary disorder. There is a disturbing increase in the incidence and prevalence of immune-mediated inflammatory diseases (IMIDs), including autoimmune and auto-inflammatory diseases. Among these, neurological conditions, such as multiple sclerosis (MS) and myasthenia gravis, are increasing at a rate of 3.7% per year while rates for gastrointestinal, endocrine, and rheumatic diseases are increasing by at least 6% per year (2). Type 1 diabetes is increasing rapidly across Europe and North America and, most disturbingly, the greatest rate of increase is in the 0- to 4-year age group (3). Similar rises in incidence rate are seen for a range of other autoimmune conditions. Crohn’s disease and ulcerative colitis, examples of auto-inflammatory conditions, are increasing at a similar rate across the world, reflecting their emergence as global diseases (4).

## Immune-Mediated Inflammatory Diseases

**Figure 1:**
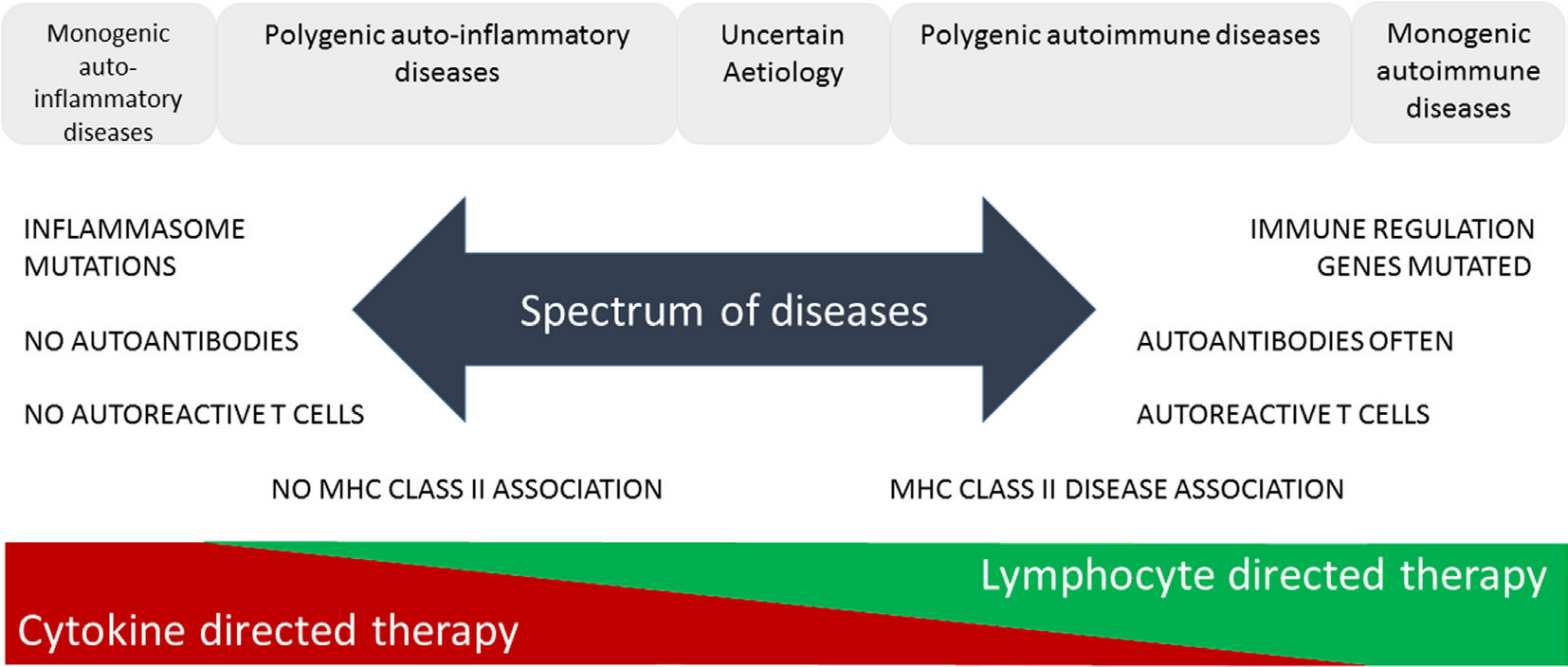
Immune-mediated inflammatory diseases. This figure is based on “A proposed classification of the immunological diseases” by McGonagle and McDermott (5). The figure distinguishes the etiological basis of auto-inflammatory and autoimmune diseases and more clearly defines the definition of these diseases according to their monogenic or polygenic basis. The spectrum of diseases proposed are more associated with either innate (auto-inflammatory) or adaptive (autoimmune) immune responses and range from monogenic auto-inflammatory to monogenic autoimmune diseases. This classification not only helps us understand the genetic and immunological basis of the disease but also predicts more effective means of immunotherapy.

The increasing prevalence of IMIDs demands a more precise classification and better fundamental understanding of the pathology underlying these diseases. This will lead to improved diagnosis through use of selective biomarkers, earlier detection and intervention thereby avoiding complications, identification of high-risk populations through better understanding of genetic and environmental influences enabling avoidance of contributory triggers or prevention through immunotherapy. McGonagle and McDermott proposed a classification of IMIDs based on the genetic factors involved in their etiology (5). They defined monogenic auto-inflammatory diseases, such as Blau syndrome, familial Mediterranean fever, and tumor necrosis factor receptor-associated periodic syndrome, as being one end of a spectrum of inflammatory diseases with monogenic autoimmune diseases, such as autoimmune lymphoproliferative syndrome, immune dysregulation polyendocrinopathy enteropathy X-linked syndrome, autoimmune polyendocrinopathy candidiasis ectodermal dystrophy, and certain complement deficiencies as the other end of the spectrum (Figure 1). The vast majority of both auto-inflammatory and autoimmune diseases, such as type 1 diabetes (6), are polygenic and, therefore, fall in between the two ends of the spectrum. Generally speaking auto-inflammatory diseases are associated with mutations influencing innate immunity, including the inflammasome genes, and are not associated with autoantibodies, autoreactive T cells or have MHC-disease associations. On the other hand, genetic polymorphisms associated with autoimmune diseases are found in genes regulating the adaptive immune system and together these permit the generation and subsequent lack of control of autoreactive T cells leading to production of autoantibodies. Strikingly, most classical autoimmune diseases have a strong association with genes in the MHC class II and are more common in women than men (7). Of the 5–10% of people in Western countries suffering from autoimmune diseases approximately 80% are women. This may be because X-chromosome inactivation or reactivation influences self-tolerance or the possibility that X-chromosome encoded miRNAs may influence susceptibility to autoimmune diseases. Furthermore, it is clear that both X-linked genes and the sex hormones produced influence innate and adaptive immunity, inflammation, and autoimmunity (7).

## Immunotherapy of Autoimmune Diseases

The Holy Grail for treatment of autoimmune diseases is to discover a means of selectively suppressing the specific autoimmune disease while leaving the rest of the immune system functionally active for control of infectious diseases and cancers. The aim is to develop treatments with increasing specificity for disease in order to decrease the risk of potential side effects. The ultimate aim is to provide a cure; however, the likelihood of success for this aim will depend on the particular autoimmune disease and associated pathology. For example, it may be sufficient to deplete autoreactive cells to correct the immune imbalance and reset homeostatic control of autoreactivity. In other cases, however, it may be necessary to continue treatment to arrest disease progression.

Currently, control of autoimmune diseases depends on the use of non-specific immunosuppressive drugs with associated side effects. Taking multiple sclerosis (MS) as an example, there are a variety of treatments that are being developed that aim to increase specificity for disease. Alemtuzumab is an antibody specific for CD52 that deletes all leukocytes and has a dramatic effect on the inflammation in and progression of MS. A median seven-year follow-up of relapsing–remitting MS patients treated with Alemtuzumab revealed that up to 70% of trial participants had an improved or unchanged disability compared to baseline. However, treatment was associated with secondary autoimmunity in approximately 48% of the treated individuals, with Graves’ disease being the most common complication (8).

Multiple sclerosis is considered to be a T-cell mediated disease; however, depletion of CD4 T cells alone was not an effective treatment (9). MS is characterized by the presence of oligoclonal bands of immunoglobulins in cerebrospinal fluid (10); however, it has been difficult to associate these antibodies with a clear target for autoimmune pathology. Nevertheless targeting CD20 on B cells is proving an effective means of controlling relapsing–remitting MS and even reducing disability in primary progressive disease (11). CD20 is expressed on pre-B, naïve, and memory B cells but not plasma cells. It is thought that depletion of these cells by rituximab or ocrelizumab will affect not only production of potentially pathogenic antibodies but also cytokine secretion by B cells and most likely their ability to present antigen to T cells (12, 13). Side effects due to B cell depletion appear limited to a higher than normal risk of herpes reactivation and breast cancer (11).

Drugs designed to reduce lymphocyte migration into the CNS have shown promising results. For example, fingolimod acts as a Sphingosine 1 phosphate (S1P) receptor agonist that results in S1P receptor downregulation thereby preventing lymphocyte migration from lymph nodes (14). This drug has a temporary effect on heart rate but otherwise has remarkably few side effects. A more targeted drug, preventing T cell migration into the CNS, is natalizumab. This drug targets the integrin VLA-4 required for lymphocytes to cross the blood brain barrier and reduces annual relapse rates and disability progression. However, there is a 1/300 chance of developing progressive multifocal leukoencephalopathy (PML) as a result of treatment (15). PML is caused by the human polyoma JC virus that infects and kills oligodendrocytes causing devastating damage when immune surveillance of the CNS is compromised.

It is difficult to see how non-specific manipulation of the immune response in autoimmune diseases such as MS will ever be completely safe or free of side effects. One increasingly promising approach is the use of low-dose interleukin 2 for treatment of autoimmune diseases (16). This relies on the fact that effector T cells respond weakly to low-dose IL-2 *in vivo* whereas Foxp3^+^ Treg cells, which express the high-affinity IL-2 receptor (CD25), proliferate following low-dose IL-2 treatment *in vivo* (17). Low-dose IL-2 treatment is well tolerated; however, it is possible that non-specific expansion of the Foxp3^+^ Treg population may influence susceptibility to infections and cancer in some individuals.

Many of the autoantigens associated with autoimmune diseases, such as MS, are known (18). In light of this, a number of groups have begun developing approaches designed to selectively target antigen-specific lymphocytes associated with autoimmune diseases. These range from injection of T-cell epitopes derived from self-antigens (19–22) through administration of tolerogenic dendritic cells carrying autoantigen peptides (23), the design of nanoparticles combined with peptide alone (24) or peptide and immunosuppressive drug (25) to the sophisticated construction of nanoparticles coated with complexes of MHC class II molecules and antigenic peptides (26, 27). Currently, the mechanisms by which these antigen-specific approaches protect against and treat autoimmune diseases are not clear. Work in preclinical models of autoimmune disease show that they function by either deleting autoreactive T cells, inducing anergy, or generating cells with a regulatory phenotype. Most importantly, results of clinical trials have not revealed significant side effects associated with antigen-specific immunotherapies.

In the next 20 years, we will discover that different regulatory T cell populations protect against different immune pathologies, including autoimmune diseases. Accordingly, we will design antigen-specific approaches optimized for induction of Foxp3^+^, IL-10-secreting Tr1-like, or CD8^+^ Treg all of which have been associated with protection from disease through antigen-specific immunotherapy. We will know how to administer antigens to selectively induce the relevant Treg population and will have tested the most effective delivery approach. Furthermore, we will have discovered drugs to co-administer with antigens in order to promote specific subsets of regulatory cells; for example, GSK-3 have been shown to promote IL-10 secreting Tr1-like cells (28) while PI3 Kinase inhibitors selectively support Foxp3^+^ Treg cells (29). Most importantly, it will be essential to identify drugs that make it possible for regulatory cells to function in an inflammatory environment (30–32).

## Immunotherapy of Cancer

Cytotoxic T cells are potent killers of cancer cells. However, both CD4 and CD8 tumor-infiltrating lymphocytes (TILs) tend to be suppressed and, hence, unable to control tumor growth. There are various mechanisms leading to suppression of TILs including the presence of Treg cells (33, 34) and the secretion of inhibitory mediators, such as adenosine, prostaglandins, and arginase (35–38). A universal feature of TILs is the upregulation of inhibitory receptors on those cells that are unable to control the cancer (39). Molecules currently under investigation include CTLA-4, PD-1, LAG-3, TIGIT, and Tim-3. The outcome of clinical trials reveals that antibodies to PD-1 and CTLA-4 are extremely powerful in reversing the suppression of TILs. Their use has shown great promise in different cancer types, prominently melanoma and small-cell lung carcinoma (40). However, the use of such “checkpoint inhibitors” does not work in all patients and we currently do not understand why. Furthermore, the use of checkpoint inhibitors, such as the combination of anti-PD-1 and anti-CTLA-4, causes severe toxicity in the majority of patients treated. Toxicity depends on the individual and ranges from inflammation of the GI tract, the most common complication, to autoimmune phenomena affecting the thyroid, skin, liver, joints, pancreas, and brain, i.e., common targets for organ-specific autoimmune diseases. At this time, we do not understand why treatment with the same combination of antibodies induces discrete autoimmune phenomena in different individuals; presumably, this reflects the presence of selective groups of pre-disposing genes in these individuals. Much current research involves investigation of altered dosing regimens or combinations of checkpoint inhibitors in order to reduce the level of toxicity. Injection of checkpoint inhibitors directly into metastatic tumor sites could enhance their efficacy with less associated toxicity as shown for Treg depleting antibodies (41). However, breaking the tolerance of TILs may never be possible without causing some degree of induced self-reactivity unless there is a means of selectively activating tumor-specific cells while leaving other self-reactive cells dormant.

**Figure 2:**
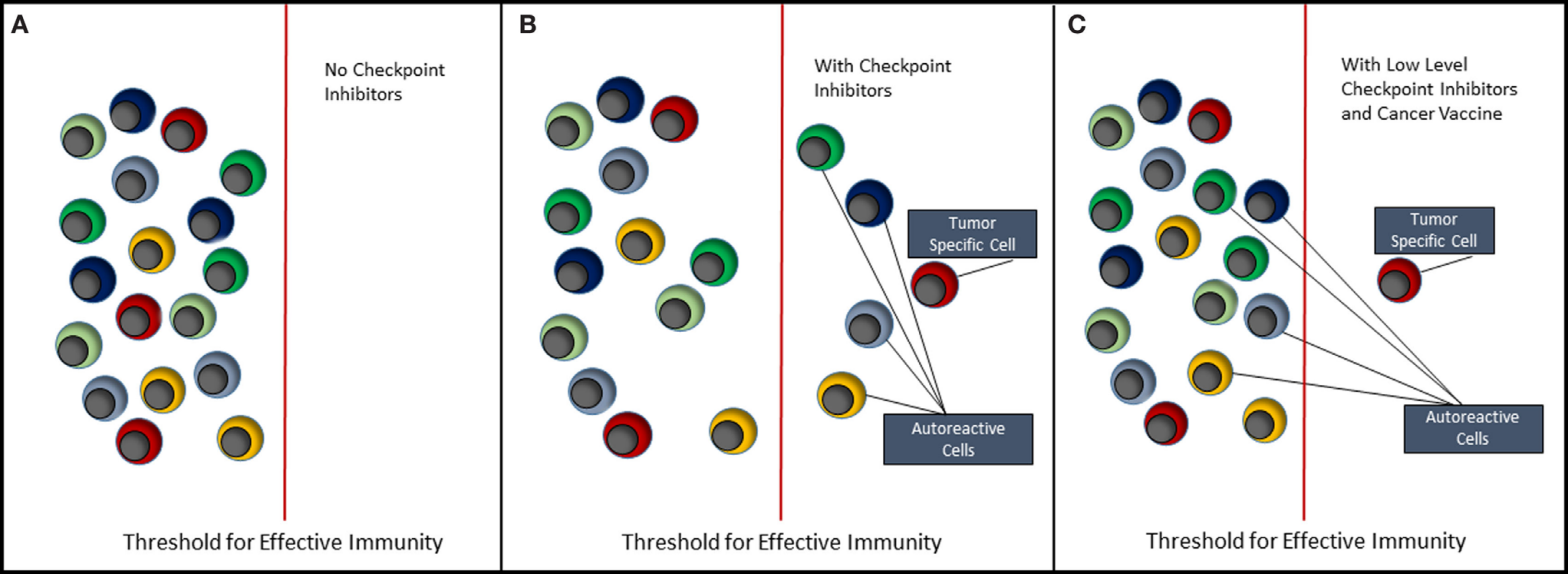
Immunotherapy of cancer. This figure depicts the effect of immunotherapy with checkpoint inhibitors on the immune system. **(A)** It reflects the steady state in which both tumor-antigen reactive and self-antigen reactive T cells remain quiescent, i.e., in a state of tolerance. **(B)** When checkpoint inhibitors such as anti-PD-1 and anti-CTLA-4 are administered, both tumor-specific and autoreactive T cells break tolerance, respond to their antigens, clear the tumor, but allow expansion of autoreactive T cells resulting in autoimmune disease. **(C)** It reflects the situation where either the type of inhibitory receptor targeted by checkpoint inhibitor is changed or the amount of checkpoint inhibitor is reduced to a level that does not trigger autoreactive T cells. It is suggested, however, that coadministration of a selective cancer vaccine will lead to expansion of tumor-specific T cells and hence tumor clearance.

The future of cancer immunotherapy lies in the combination of selective cancer vaccines and checkpoint inhibitors or some other means of relieving immune suppression associated with the tumor. As shown in Figure 2, it is possible to lower the threshold for effective antitumor immunity by blocking inhibitory receptors such as PD-1 and CTLA-4. However, the use of checkpoint inhibitors alone (Figure 2B) will never selectively activate tumor-specific cells without coincidentally causing activation of self-reactive cells and, hence, causing some form of auto-inflammatory or autoimmune condition. Currently, we understand very little about how most of the inhibitor receptors targeted by checkpoint inhibitors actually function (42). These molecules downregulate cell signaling at the immune synapse; however, the mechanisms involved are largely unknown. Detailed knowledge of this would reveal common signaling and regulatory pathways that could provide more controlled targets for pharmaceutical intervention. Ultimately, it should be possible to reduce the level of or change the combination of checkpoint inhibitors, such that self-reactive cells are no longer activated. We then need a means of selectively immunizing for an antitumor response using a cancer vaccine.

Importantly, we are entering a revolutionary era for research into the neoantigens associated with tumors and the application of this knowledge in vaccine development. For example, the use of massively parallel sequencing for detection of mutations within tumors combined with machine learning approaches, to predict which of those mutated peptides bind with high affinity to HLA molecules, has allowed development of immunogenic vaccines targeting predicted neoantigens. A recent study described the application of this approach in melanoma whereby four of six vaccinated patients had no recurrence 2 years after vaccination while two with recurrent disease experienced complete tumor regression following treatment with anti-PD-1 (43). This outstanding achievement, combining molecular analysis and computer prediction, holds great promise for the future of cancer vaccination and shows the power of combination immunotherapies. The same combination approach could be applied to the tumor microenvironment whereby inhibition of suppressive molecules, such as adenosine, prostaglandin, or arginase, could be combined with a vaccine to boost the anti-cancer approach. The next 20 years will see a stream of breakthroughs in which immunotherapeutic approaches are combined to selectively target tumors while avoiding unnecessary toxicities.

## Conclusion

The immune system has evolved to protect us from infection. The human immune system is immensely complex and the drawback of developing an immune system that may recognize and respond to all infections is the potential for hypersensitivity reactions. These manifest as allergic responses to environmental agents and autoimmune responses to self-antigens. Equally, the immune system has developed sophisticated regulatory mechanisms to protect against rejection of the human allograft during pregnancy and reduce the risk of autoimmune diseases. These immune regulatory mechanisms serve as barriers to effective cancer immunity: the challenge to cancer control and eradication is how to have one without the other, i.e., how to promote effective cancer immunity without the toxic side effects of autoimmune diseases. Recent breakthroughs in the use of checkpoint inhibition, when combined with cancer vaccination, will make this feasible: the key factor is to target the relevant cancer antigen. For autoimmune diseases, we have depended on non-specific immunosuppressive drugs for far too long. We have failed to learn from the allergy field where effective immunotherapy is achieved by targeted desensitization using allergy associated antigens. The antigen-specific immunotherapies referred to in this perspective article herald a new era of immunotherapy for autoimmune diseases where again the key factor is to target the relevant antigen, in this case the self-antigen.

## Author Contributions

DW conceived and wrote the perspective article.

## Conflict of Interest Statement

DW is CSO of Apitope International. Apitope develops antigen-specific immunotherapies for autoimmune diseases.
